# Relationship between chemokine receptor expression, chemokine levels and HIV-1 replication in the lungs of persons exposed to *Mycobacterium tuberculosis*

**DOI:** 10.1002/eji.201242804

**Published:** 2012-12-13

**Authors:** Barbara Kalsdorf, Keira H Skolimowska, Thomas J Scriba, Rod Dawson, Keertan Dheda, Kathryn Wood, Jessica Hofmeister, Willem A Hanekom, Christoph Lange, Robert J Wilkinson

**Affiliations:** 1 Clinical Infectious Diseases Research Initiative, Institute of Infectious Diseases and Molecular Medicine, University of Cape TownObservatory, Cape Town, South Africa; 2 Clinical Infectious Diseases, Tuberculosis Center, Research Center BorstelBorstel, Germany; 3 Department of Medicine, Imperial College LondonLondon, UK; 4 South African Tuberculosis Vaccine Initiative and School of Child and Adolescent Health, Institute of Infectious Diseases and Molecular Medicine, University of Cape TownObservatory, Cape Town, South Africa; 5 Department of Medicine, University of Cape TownObservatory, Cape Town, South Africa; 6 University of Schleswig Holstein, Campus LübeckLübeck, Germany; 7 MRC National Institute for Medical ResearchLondon, UK

**Keywords:** BAL, CCR5, RANTES, TB, Viral load

## Abstract

Increased susceptibility to tuberculosis following HIV-1 seroconversion contributes significantly to the tuberculosis epidemic in sub-Saharan Africa. Lung-specific mechanisms underlying the interaction between HIV-1 and *Mycobacterium tuberculosis* infection are incompletely understood. Here we address these questions by examining the effect of HIV-1 and latent *M. tuberculosis* co-infection on the expression of viral-entry receptors and ligands in bronchoalveolar lavage (BAL) of HIV-1-infected and -uninfected patients with and without latent *M. tuberculosis* infection. Irrespective of HIV-1 status, T cells from BAL expressed higher levels of the beta-chemokine receptor (CCR)5 than peripheral blood T cells, in particular the CD8^+^ T cells of HIV-1-infected persons showed elevated CCR5 expression. The concentrations of the CCR5 ligands RANTES and MIP-1β were elevated in the BAL of HIV-1-infected persons compared with that in HIV-1-uninfected controls. CCR5 expression and RANTES concentration correlated strongly with HIV-1 viral load in the BAL. In contrast, these alterations were not associated with *M. tuberculosis* sensitisation in vivo, nor did *M. tuberculosis* infection of BAL cells ex vivo change RANTES expression. These data suggest ongoing HIV-1 replication predominantly drives local pulmonary CCR5^+^ T-cell activation in HIV/latent *M. tuberculosis* co-infection.

## Introduction

Tuberculosis and AIDS are amongst the leading causes of death worldwide. In the majority of cases in immunocompetent persons, *Mycobacterium tuberculosis* is successfully controlled by local immune responses leading to isolation of infected alveolar macrophages and the formation of granulomas. In HIV-1 infection, the rate of active tuberculosis following primary infection and the rate of reactivation of tuberculosis in individuals with latent *M. tuberculosis* infection is greatly increased although the underlying mechanisms are not well understood 1.

T-cell mediated immunity is critical for immune control of tuberculosis. HIV-1 replicates in activated CD4^+^ T cells, monocytes or dendritic cells and leads to immunodeficiency characterised by progressive CD4^+^ T-cell depletion. Moreover, despite relatively normal numbers of circulating CD4^+^ T cells HIV-1-infected persons are already at increased risk of tuberculosis in the first year following seroconversion 2. HIV-1 infection impairs not only the quantity but also the quality of *M. tuberculosis*-specific immune responses 3. In addition mortality remains higher after successful anti-tuberculosis treatment of HIV-1-infected persons 4, even though CD4^+^ T-cell numbers partially reconstitute after initiation of anti-retroviral therapy (ART) 5.

T-cell activation is an important mechanism of HIV-1 pathogenesis 6 and persistent cell expression of activation markers predicts the progression into AIDS 7. So far only a few studies have investigated the activation markers CD38, CD69 and Ki67 on BAL cells at the site of active tuberculosis disease 6, 8, 9. Chronic antigen exposure in latent *M. tuberculosis* infection may lead to a persistent localised immune activation that facilitates HIV-1 entry into CD4^+^ T cells in lungs. The alpha (CXCR)4 and beta chemokine receptors (CCR)5 are the most important coreceptors for HIV-1 entry and infection of CD4^+^ T cells 10, 11. CCR5 expression on CD8^+^ T cells mediates the migration of antigen-specific effector and differentiated memory CD8^+^ T cells to the site of inflammation and it has been suggested that these CD8^+^ T cells are important in eradication of virus-infected CD4^+^ T cells. The ß-chemokines MIP1α, MIP1β and RANTES attract CCR5^+^ T cells to the region 12.

To evaluate the hypothesis that HIV-1 infection or *M. tuberculosis* exposure influence CCR5 and CXCR4 receptor and agonist expression, receptor expression and chemokine profiles from the peripheral blood and BAL mononuclear cells were compared between in HIV-1-infected and -uninfected persons with and without evidence of *M. tuberculosis* sensitisation.

## Results

### Participants

Blood and BAL cells from age- and sex-matched groups of 15 HIV-1-infected and 21 HIV-1-uninfected persons from an area of high tuberculosis incidence and high HIV-1 prevalence were investigated 3 (Table 1). Ten HIV-1-infected and 11 HIV-1-uninfected participants were diagnosed with latent *M. tuberculosis* infection by a positive ESAT-6 and/or CFP-10 specific IFN-γ immune response in an ELISPOT assay performed with PBMCs in the absence of active tuberculosis disease. HIV-1-infected persons had a median CD4^+^ count of 226 cells/μL. Frequencies of CD4^+^ CD3^+^ T cells were significantly lower in blood (median 10.0 versus 42.4%, *p* < 0.001) and BAL lymphocytes (median 7.7 versus 37.7%, *p* < 0.001) when compared with those in HIV-1-uninfected persons (Table 1).

**Table 1:** Characteristics of persons enrolled to the study

	HIV-1 infected	HIV-1 uninfected	*p*-value
N	15	21	
Sex: female/male, *n*	11/4	13/8	0.564
Age, mean years (range)	34.3 (24–51)	32.7 (21–55)	0.531
CD4, median CD4 cells/μL (range)	226 (61–595)	786 (461–1225)	<0.001a)
Blood CD4^+^, median CD4^+^ as % of CD3^+^ T cells (range)	10.0 (3.45–28.9)	42.4 (9.46–67.8)	<0.001a)
BAL CD4^+^, median CD4^+^ as % of CD3^+^ T cells (range)	7.7 (1.33–31.30)	37.7 (12.5–78.7)	<0.001a)
Blood viral load, median RNA copies/mL, (range)	22 000 (590–660 000)		
BAL viral load, median RNA copies/mL, (range)	1964 (855–709 088)		
Response to ESAT-6/CFP-10, blood: positive/negative	10/5	11/10	0.190b)

a) Differences between the HIV-1-infected and -uninfected groups were compared using the Mann–Whitney U-test.

b) Difference of ESAT-6/CFP-10-induced IFNγ-response in PBMCs between HIV-1-infected and -uninfected groups was compared using Fisher’s exact test of probability.

### HIV-1 viral load

**Figure 1:**
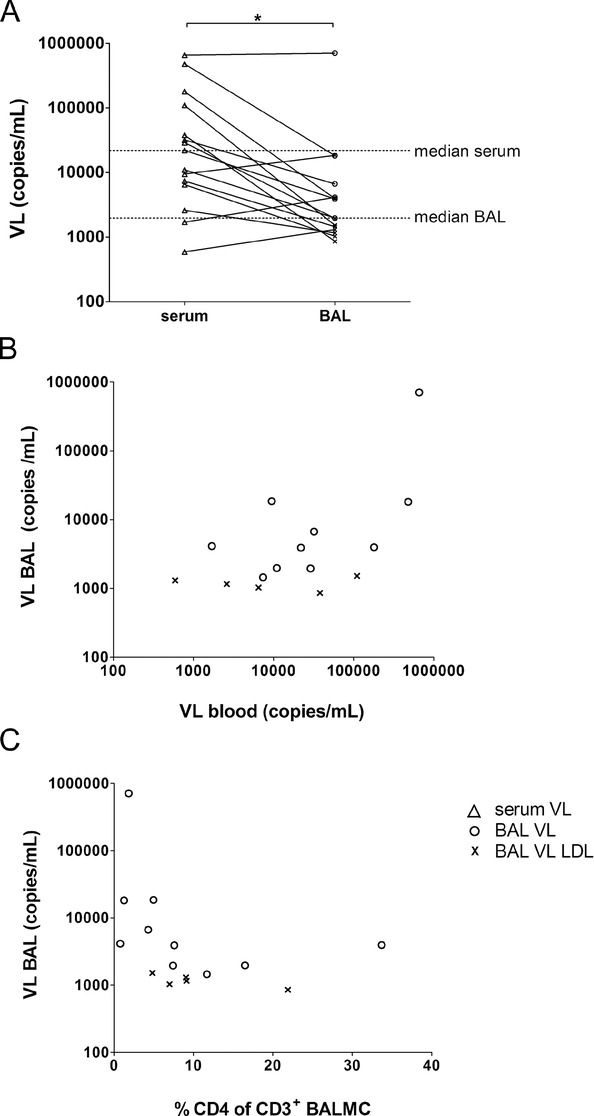
Viral load (VL) in bronchoalveolar lavage (BAL). (A) Paired VL in serum (*n* = 15, open triangles) and BAL fluid (*n* = 15) was measured by nucleic acid amplification tests of *gag* for the quantitation of human HIV-1 RNA. The detection limit was 20 copies/800 μL of pure BAL fluid. BAL VLs above the detection limit are depicted with open circles (*n* = 10). BAL VL values lower the detection limit (LDL) were set to a value of 19 copies/mL and normalised according to the urea method (*n* = 5, symbol ×). Each symbol represents an individual sample, horizontal lines represent median VL of serum and BAL. *p = 0.030 between serum and BAL VL, Wilcoxon signed rank test. (B) Correlation between VL in serum and BAL was assessed by Pearson (*ρ* = 0.787, *p* < 0.001) and (C) correlation between VL BAL and the relative frequency of CD4^+^ CD3^+^ BAL T cells was assessed by Spearman (*ρ* = −0.586, *p* = 0.022). Data shown are pooled data from 15 experiments performed.

Stratification of BAL urea level by HIV-1 status revealed no difference between HIV-1-uninfected and -infected people (*p* = 0.64, data not shown). To compare the tissue load of HIV-1 in the lungs with the levels in blood, the viral load was determined in serum and BAL (Fig. 1A). In 5 out of 15 HIV-1-infected persons the viral load in BAL was below the detection limit (individuals marked in the graphs). The median viral load in serum was 22 000 RNA copies/mL (range 590–660 000 copies/mL). A significantly lower median of 1964 RNA copies/mL (range 855–709 088 copies/mL) was found in BAL (*p* = 0.030). Nevertheless, in three cases the viral load in the BAL was higher than the viral load in serum. There was a positive correlation between the viral load in serum and in lungs (*ρ* = 0.787, *p* < 0.001, Fig. 1B). Figure 1C demonstrates an inverse correlation (*ρ* = −0.586, *p* = 0.022) between viral load and the proportion of CD4^+^ in BAL.

### HIV-1 receptor expression on bronchoalveolar T cells

**Figure 2:**
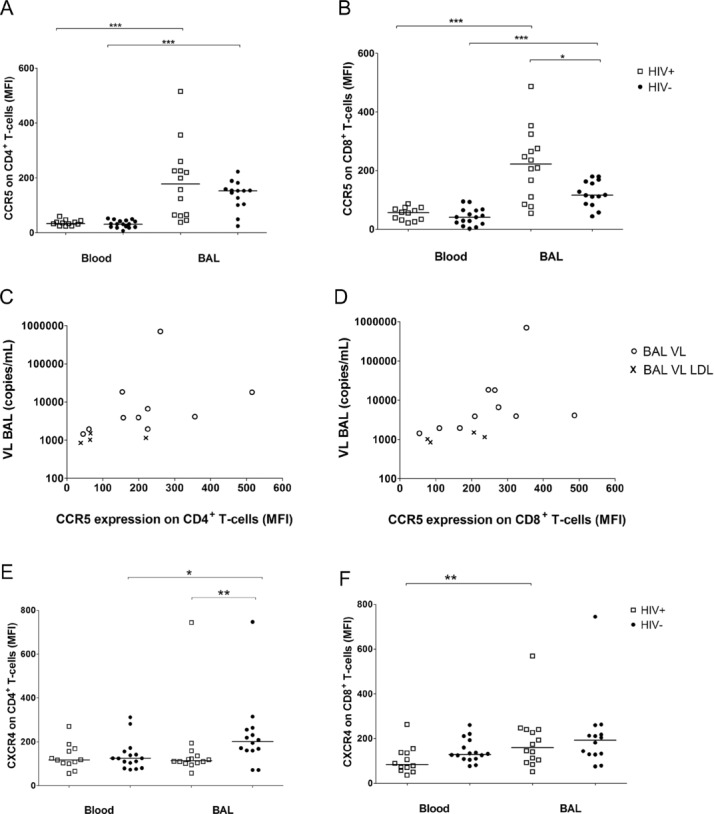
CCR5 and CXCR4 expression on CD4^+^ or CD8^+^ T cells in blood and BAL. (A, B, E, F) The median fluorescence intensity (MFI) of (A, B) CCR5 and (E, F) CXCR4 receptor expression was measured by flow cytometry on (A, E) CD4^+^ and (B, F) CD8^+^ cells from the blood and BAL of HIV-1-infected (*n* = 12 in blood, *n* = 14 in BAL, open squares) and HIV-1-uninfected persons (*n* = 16 in blood, *n* = 14 in BAL, solid circles), each sign represents one individual, bars represent medians. Differences between CCR5 expression on CD4^+^ and CD8^+^ T cells from paired blood and BAL samples were calculated by Wilcoxon signed rank test (***p < 0.001). The CCR5 expression on CD8^+^ T cells in the BAL was significantly higher in HIV-1-infected compared to HIV-1-uninfected persons (*p = 0.026, by Mann–Whitney U-test). (C, D) The correlation between viral load (VL) in BAL and MFI of (C) CCR5^+^ on CD4^+^ (*ρ* = 0.706, *p* = 0.005) or (D) CD8^+^ (*ρ* = 0.793, *p* < 0.001) BAL T cells of HIV-1-infected participants was assessed by Spearman. Open circles, BAL VL; symbol ×, BAL VL LDL were set to a value of 19 copies/mL and normalised according to the urea method. (E) The difference of CXCR4 expression on CD4^+^ paired blood and BAL T cells was measured by Wilcoxon signed rank test (*p = 0.049). MFI of CXCR4^+^ CD4^+^ BALMCs was significantly higher in the HIV-1-uninfected control group when compared with that of HIV-1-infected persons (**p = 0.009, Mann–Whitney U-test). (F) BAL CD8^+^ T cells expressed higher levels of CXCR4 than PBMCs in HIV-1-infected persons (**p = 0.003), differences between the HIV-1 status were assessed by Mann–Whitney U-test. Data shown are pooled data from experiments on 12 blood and 14 BAL samples of HIV-1-infected and 16 blood and 14 BAL samples of HIV-1-uninfected persons.

Several mechanisms of increased HIV-1 replication have been described at the site of *M. tuberculosis* infection 13. In the previous study 3 which was performed with the same participants, no differences were found in the T-cell memory phenotype between HIV-1-infected and -uninfected persons, but in both groups there was a shift to the predominance of CD4^+^ effector T cells in BAL when compared with blood. As these activated effector T cells in the lungs may facilitate HIV-1 entry, BAL mononuclear cells (BALMCs) were characterised for their expression of the HIV-1 entry receptors CCR5 and CXCR4. Comparing the compartments, CD4^+^ (Fig. 2A) and CD8^+^ BALMCs (Fig. 2B) invariably had significantly higher expression of the CCR5 receptor than Peripheral blood mononuclear cells (PBMCs) (*p* < 0.001), measured by median fluorescence intensity (MFI). No differences in CCR5 expression by CD4^+^ blood T cells were found between HIV-1-infected and -uninfected persons, whereas CCR5 expression by CD4^+^ T cells in BAL was slightly higher in HIV-1-infected persons than in HIV-1-uninfected participants (178 versus 153) although the difference was not significant (*p* = 0.37, Fig. 2A). Similarly, CCR5 expression by peripheral CD8^+^ T cells did not differ between HIV-1-infected and -uninfected persons. In contrast, CCR5^+^ expression by CD8^+^ T cells in BAL was moderately higher in HIV-1-infected persons (222.5 versus 116.5, *p* = 0.026, Fig. 2B). Levels of CCR5 on CD4^+^ and CD8^+^ BAL T cells were directly correlated with viral load in BAL (*ρ* = 0.706, *p* = 0.005, Fig. 2C and *ρ* = 0.793, *p* < 0.001, Fig. 2D). In contrast, stratifying CCR5 expression on CD4^+^ and CD8^+^ BALMCs by *M. tuberculosis* infection status did not reveal any difference (data not shown).

The pattern of CXCR4 expression on T cells differed from CCR5 expression. Levels of CXCR4 expression on CD4^+^ T cells trended to be higher on BALMCs than on PBMCs in HIV-1-uninfected subjects (201.5 versus 124.5, *p* = 0.049), whereas no difference in CXCR4 expression was observed between BAL or blood CD4^+^ T cells of HIV-1-infected persons. CXCR4 expression on CD4^+^ BALMCs was significantly higher in the HIV-1-uninfected control group when compared with that of HIV-1-infected persons (201.5 versus 114.5, *p* = 0.009, Fig. 2E). No correlation was found between CXCR4 expression by CD4^+^ BALMCs and viral load in BAL in HIV-1-infected persons (data not shown).

CXCR4^+^ expression on CD8^+^ PBMCs did not differ by HIV-1 status. BALMCs CD8^+^ T cells expressed higher levels of CXCR4^+^ than PBMCs in HIV-1-infected persons (159.5 versus 83.9, respectively, *p* = 0.003, Fig. 2F), whereas the slightly higher CXCR4 expression on CD8^+^ BAL T cells compared with that of PBMCs in the HIV-1-uninfected group was not significant (193 versus 129.5, *p* = 0.059).

### Level of CCR5 ligands

**Figure 3:**
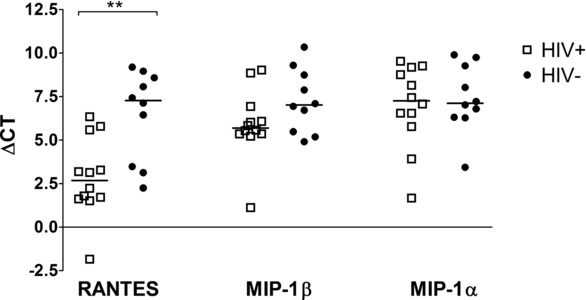
Constitutive transcript abundance of CCR5 ligands in BAL cells. Constitutive transcript abundance was assessed in freshly isolated BALMCs of HIV-1-infected persons (*n* = 12, open squares) and HIV-1-uninfected persons (*n* = 10, solid circles). ΔCT = (CT gene of interest) − (CT β-actin). A lower ΔCT indicates higher transcript abundance. Each symbol represents one sample, bars represent medians. Differences between constitutive RANTES transcript abundance of HIV-1-infected and -uninfected persons was determined by the Mann–Whitney U-test (**p = 0.003). Data shown are pooled data from experiments on 12 HIV-1-infected and 10 HIV-1-uninfected persons.

Blockade of the CCR5 receptor by drugs, such as Maraviroc, can prevent the entry of CCR5-tropic HIV-1 into target cells 14, 15. As differences were observed in the expression of CCR5 in BAL compared with that in blood, CCR5 ligand levels were ascertained in the two compartments by measuring the transcript levels of the three CCR5 agonists RANTES, MIP-1β and MIP-1α in BALMCs. A lower threshold cycle (ΔCT) represents a higher abundance (Fig. 3). RANTES abundance was significantly higher in HIV-1-infected than in uninfected participants (median ΔCT 2.68 versus 7.27, *p* = 0.003). The mean abundance of MIP-1β was slightly but not significantly higher in HIV-1-infected (median ΔCT 5.70), when compared with that of HIV-1-uninfected persons (median ΔCT 7.02, *p* = 0.225), whereas MIP-1α transcript abundance in BAL was comparable in HIV-1-infected and uninfected persons.

**Figure 4:**
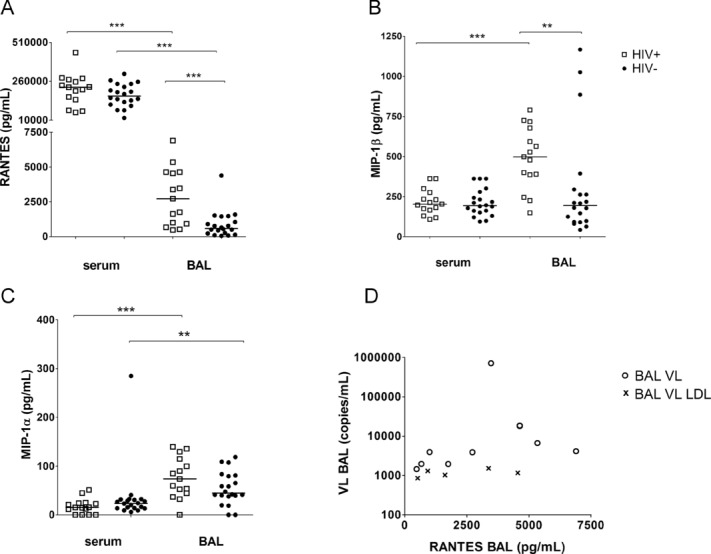
Chemokine concentration of RANTES, MIP-1β and MIP-1α in serum and BAL of HIV-1-infected (*n* = 15, open squares) or HIV-1-uninfected (*n* = 20, solid circles) persons. Chemokine concentration was measured by multiplex bead array; each symbol represents one sample, bars represent medians. (A) Differences of RANTES concentration between paired serum and BAL samples of HIV-1-infected (***p < 0.001) and HIV-1-uninfected persons were assessed by Wilcoxon signed rank test. The RANTES level in BAL in HIV-1-infected in comparison with HIV-1-uninfected (***p < 0.001) was calculated by Mann–Whitney U-test. (B) Difference of MIP-1β concentration between paired serum and BAL samples of HIV-1-infected participants (***p < 0.001) was assesses by Wilcoxon signed rank test. MIP-1β level in BAL was higher in HIV-1-infected versus HIV-1-uninfected persons (**p = 0.004, Mann–Whitney U-test). (C) Wilcoxon signed rank test was used to compare MIP-1α level in paired blood and BAL samples (***p < 0.001 in HIV-1-infected, **p = 0.01 in HIV-1-uninfected persons). (D) Correlation between RANTES concentration in BAL and viral load (VL) in BAL were assessed by Spearman (*ρ* = 0.635, *p* = 0.011). Open circles, BAL VL; symbol ×, BAL VL LDL were set to a value of 19 copies/mL and normalised according to the urea method. Data shown are pooled data from experiments on 15 HIV-1-infected and 20 HIV-1-uninfected persons.

Having observed differences in transcript abundance, the protein levels of RANTES, MIP-1β and MIP-1α were investigated by multiplex bead array in blood and BAL. RANTES was found at high concentrations in serum, but at significantly lower concentrations in the BAL (*p* < 0.001). The level of this chemokine in serum was independent of HIV-1 status, whereas significantly higher RANTES levels were recorded in the BAL of HIV-1-infected versus HIV-1-uninfected persons (2725 pg/mL versus 594.6 pg/mL, *p* < 0.001, Fig. 4A). The RANTES levels in BAL also correlated with viral load in the same compartment (*ρ* = 0.635, *p* = 0.011, Fig. 4D), whereas elevated RANTES levels were not associated with *M. tuberculosis* sensitisation status (data not shown).

MIP-1β levels in blood were comparable in HIV-1-uninfected and -infected persons. In contrast, MIP-1β was elevated in the BAL of HIV-1-infected compared with that of HIV-1-uninfected persons (497.2 pg/mL versus 194.3 pg/mL, *p* = 0.004). Furthermore, MIP-1β levels in the BAL of HIV-1-infected participants were significantly higher when compared with that of the blood compartment (497.2 pg/mL versus 202.8 pg/mL, *p* < 0.001, Fig. 4B).

Only low concentrations of MIP-1α were detected in both blood and serum. The serum levels of MIP-1α in HIV-1-infected participants did not differ from uninfected persons. The MIP-1α level in the BAL tended to be slightly higher in HIV-1-infected versus uninfected persons (73.7 pg/mL versus 44.7 pg/mL, *p* = 0.113). Comparing both, blood and BAL compartments, significantly higher levels of MIP-1α were found in the BAL compared with that in the serum of HIV-1-infected (73.7 pg/mL versus 15.2 pg/mL, respectively, *p* < 0.001) and HIV-1-uninfected participants (44.7 pg/mL versus 23.5 pg/mL, *p* = 0.01, Fig. 4C).

No difference in the concentration of CXCR4 ligand stromal cell-derived factor 1 (SDF-1α) in serum was observed between HIV-1-infected and -uninfected persons. Levels of SDF-1α in BAL samples were all below the detection limit of 18.9 pg/mL (data not shown).

### Effect of *M. tuberculosis* infection on CCR5 ligand expression

**Figure 5:**
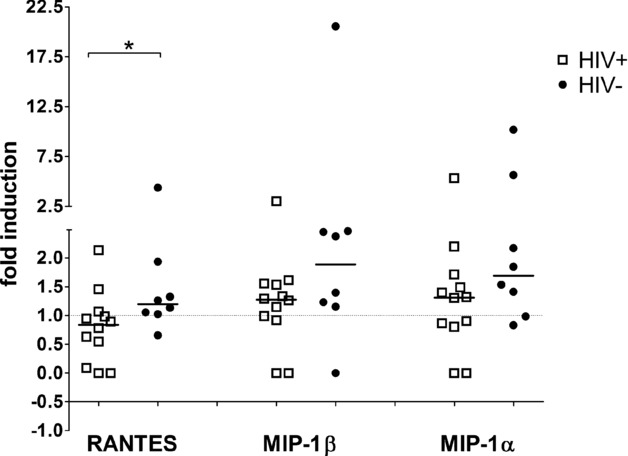
Fold induction of CCR5 ligands in bronchoalveolar lavage cells after 24 h *M. tuberculosis* stimulation. 5 × 10^5^ BALMCs of HIV-1-infected (*n* = 12, open squares) and HIV-1-uninfected participants (*n* = 8, solid circles) were cultured for 24 h in the presence or absence of *M. tuberculosis* H37Rv at MOI 1:1. Fold induction was calculated as (ΔCT in the presence of H37Rv) − (ΔCT in the absence of H37Rv), values normalised by power 2^×^ transformation. Each symbol is representative of one sample, bars represent medians. Differences between RANTES constitutive transcript abundance of HIV-1-infected and -uninfected persons (*p = 0.034) were assessed by Mann–Whitney test. Data shown are pooled data from experiments on 12 HIV-1-infected and 8 HIV-1-uninfected persons.

To investigate if differences in CCR5 ligand expression were due to HIV-1 infection or *M. tuberculosis,* BALMCs were cultured in the presence or absence of *M. tuberculosis* H37Rv for 24 h and transcript abundance measured by RT-PCR. Incubation with H37Rv decreased RANTES expression in BALMCs from HIV-1-infected persons (median 0.84-fold induction), BALMCs of HIV-1-uninfected persons showed a slight increase in RANTES expression (median 1.20-fold, *p* = 0.034, the difference is within experimental error). H37Rv induced little increase in MIP-1β (medians 1.28-fold in HIV-1-infected in comparison to 1.89-fold in HIV-1-uninfected). Similarly, median fold increases of 1.31 and 1.69 were observed in MIP-1α transcripts in HIV-1-infected and -uninfected persons, respectively. Therefore the influence of *M. tuberculosis* on the gene expression level of CCR5 agonists was not great, with RANTES gene expression even diminished in HIV-1-infected persons (Fig. 5).

## Discussion

The immunological mechanisms underlying the increased risk of tuberculosis in persons with HIV-1 co-infection, and those that exazerbate in the course of both diseases, are unclear. The aim of this study was to investigate the influence of HIV-1 infection on immune activation of resident bronchopulmonary lymphocytes and a possible permissive environment for HIV-1 entry and replication. Therefore, chemokine receptors and chemokine profiles of the two compartments lavage and blood of HIV-1-infected and -uninfected persons from an area of high tuberculosis exposure were compared. The key findings of this study are: (i) viral load correlates inversely with the number of CD4^+^ T cells in BAL, (ii) CD8^+^ BAL T cells of HIV-1-infected persons express higher levels of CCR5 compared with HIV-1-uninfected persons, and (iii) the observed elevation of MIP-1β and RANTES levels in BAL of HIV-1-infected persons was not further upregulated by ex vivo *M. tuberculosis* infection.

In this study HIV-1 viral load in BAL correlated directly with viral load in serum and inversely with CD4^+^ cell count in BAL. These data support previous observations of HIV-1 replication in lungs 16, 17. Previous studies have demonstrated that HIV-1 replicates in alveolar macrophages 18, 19, others showed that CCR5^+^ CD4^+^ BAL T cells support replication 5, 20. These results show in some people an elevation of HIV-1 RNA concentration in BAL fluid compared to serum, which supports compartmentalised local viral replication in human lungs. Furthermore, lung segments affected by tuberculosis disease have significantly higher HIV-1 viral load than uninvolved paired lung segment 21. This may be partially explained by the finding that activated T cells display greater permissiveness to infection and viral replication, compared with quiescent cells 22. To this end, HIV-1 replication was recently associated with *M. tuberculosis*-specific CD4^+^ T cells, compared with CMV-specific CD4^+^ T cells 23.

This study compared expression of the HIV-1 entry receptors on BAL and blood T cells of HIV-1-infected and -uninfected persons living in an area of high tuberculosis incidence. There was a wide range of CXCR4 expression levels on T cells, with significantly decreased levels of CXCR4^+^ on CD4^+^ BAL T cells of HIV-1-infected compared with those of HIV-1-uninfected persons. However, CXCR4 expression by CD4^+^ BALMCs was not associated with HIV-1 viral load in the same compartment, confirming previous findings 24.

Higher levels of CCR5 expression were observed on CD4^+^ BAL T cells by comparison with peripheral blood cells. This may be due to the fact that CCR5 is mainly expressed on effector memory (CD45RA^−^ and CD27^−^) T cell 25, the predominant T-cell subset in BAL 3. As CCR5 expression increases permissiveness to HIV-1 infection 11, the elevated expression of CCR5 on CD4^+^ BAL T cells might increase BALMC susceptibility to HIV-1 infection. Our finding that HIV-1 viral load correlated with CCR5 expression on CD4^+^ BAL T cells supports this hypothesis. Therefore these data add to previous descriptions of BALMCs expression of CCR5 5, 9, 20, 26 at the site of *M. tuberculosis* infection.

Findings from this study suggest that HIV-1 infection induces the elevation of CCR5^+^ expression on CD8^+^ BAL T cells. Although increased expression of CCR5 on CD8^+^ T cells is not directly related to HIV-1 susceptibility, CD8^+^ T cells play a critical role in the control of viral infection. During HIV-1 infection cytotoxic T-lympocytes eliminate HIV-1-infected cells and secrete β-chemokines 27, which recruit monocytes and T cells to inflammatory sites. Because our results showed an increased presence of CD8^+^ T cells at the site of infection, we expected to find an elevation of the β-chemokines RANTES, MIP-1β and MIP-1α in BAL. This hypothesis was confirmed as higher levels of RANTES and MIP-1β were found in the BAL of HIV-1-infected in comparison to HIV-1-uninfected persons. It should be noted that soluble chemokines can be used to determine relative changes of these peptides during an inflammatory process but may more represent evidence of inflammation than a biological relevant form of these mediators. Since minimal concentrations of RANTES required for activation of CCR5 on T cells are between 1 and 10 nM 28, about threefold lower levels found in BAL derived from HIV-infected patient (2700 pg/mL; ∼0.35 nM) are unlikely able to modulate the receptor status, but indicate to a higher status of cell activation in the lungs of HIV-1-infected persons.

RANTES levels directly correlated with viral load in BAL. On restimulation of BAL cells with *M. tuberculosis* no upregulation of the RANTES gene expression was observed. This suggests that CCR5 agonist levels in BALMCs are influenced rather by HIV-1 status than by *M. tuberculosis* infection. Regulation of β-chemokine expression in BAL cells may differ from peripheral blood cells, as the latter showed higher β-chemokine levels in active tuberculosis, compared with healthy controls 29 and peripheral levels remained elevated in HIV-1 and *M. tuberculosis* coinfected persons despite anti-tuberculosis treatment 30.

HIV-1 viral replication in the lungs may drive the production of MIP-1β and RANTES. We speculate that this, at least in part, reflects compartmentalised cytolytic activity of HIV-1-specific CD8^+^ T cells 31, 32. Circulating T cells that have been activated by β-chemokines and migrate to the site of infection, may thus be preferentially infected by HIV-1 33. By contrast, locally primed antigen-specific T cells are less permissive to HIV-1 infection, as CCR5 agonists compete with HIV-1 for binding sites of the CCR5 receptor resulting in an inhibitory effect for HIV-1 entry 14, 30, 33.

Our study had several limitations. Due to restricted numbers of BAL cells we were unable to better characterise the CCR5^+^ T-cell populations in respect of memory or effector phenotyping and to investigate the HIV-1-specific immune response. Such investigations would have allowed us to address whether, as shown for peripheral blood cells, antigen-specific CD4^+^ BAL T cells that express high levels of CCR5 are preferentially eliminated 34 or if ongoing viral replication would be a similar predictor of HIV-1-specific CD8^+^ T-cell loss in BAL 35. Secondly, it might be possible that HIV-1 infection preferentially depletes *M. tuberculosis*-specific CCR5^+^ CD4^+^ T cells 9, 23, 36. Although we have not addressed this question specifically, data from the same participants show that *M. tuberculosis*-specific T-cell responses in lungs of HIV-1-infected persons are markedly impaired 3. It will be important to also study these effects of HIV infection in patients with active pulmonary tuberculosis.

## Conclusion

Elevated levels of CCR5, the coreceptor for HIV-1, on CD4^+^ BAL T cells in comparison to peripheral blood cells suggest a local permissive environment for HIV-1 infection in human lungs. HIV-1-infected persons also exhibited higher expression of CCR5 by CD8^+^ BAL T cells, suggesting that CCR5 may play an important role in the recruitment of HIV-1-specific CD8^+^ effector T cells into inflamed tissue where these CD8^+^ T cells may mediate killing of HIV-infected cells. These results provide further evidence that ongoing HIV-1 replication is an important factor in the elevation of MIP-1β and RANTES levels in BAL from persons with HIV/latent *M. tuberculosis* co-infection.

## Materials and methods

### Participants

The study was approved by the Research Ethics Committees of the Universities of Cape Town, South Africa (REC 381/2006), and Lübeck, Germany (05-096) and all participants provided written informed consent. These investigations were performed as part of a larger study. Cells from the same patient samples were used for different experiments, these results focussing on the influence of HIV-1 on *M. tuberculosis-*specific T-cell immune responses have been previously published 3.

Briefly, participants were recruited at the Khayelitsha Site B Clinic in Cape Town, South Africa. In compliance with South African national guidelines HIV-1 care including ART was offered to all HIV-1-infected persons. A symptom screen and physical examination were performed, persons with active or a past history of tuberculosis, or isoniazid preventive therapy were excluded. Smoking, pregnancy, chronic cardiovascular or metabolic illnesses, immunosuppressive medication, and age less than 21 years also constituted exclusions. All participants had negative cultures for *M. tuberculosis* in BAL and had no radiological evidence of lung disease.

### BAL and blood collection and processing

Bronchoscopy and blood collection were performed before patients were initiated on ART. Standard flexible diagnostic bronchoscopy including a BAL of the middle lobe with 300 mL sterile saline and isolation of the bronchoalveolar mononuclear cells (BALMCs) were conducted as described previously 3, 37. BAL fluid was harvested and aliquots were frozen immediately at −80°C. PBMCs were prepared as described previously 3, 37. Serum samples were centrifugated at 3000 rpm for 15 min at 20°C, aliquoted and frozen at −80°C.

### Latent *M. tuberculosis* infection

Sensitisation by *M. tuberculosis* was defined by the immune responses to the *M. tuberculosis-*specific antigens early secreted antigenic target (ESAT)-6 and culture filtrate protein (CFP)-10 measured in PBMCs by IFN-γ ELISPOT assay, MABTECH (Nacka, Sweden) as reported previously 3.

### HIV-1 viral load

Plasma HIV-1 viral load was detected by Nuclisens (BioMerieux, Randburg, South Africa). Viral load in BAL fluid was measured by Cobas TaqMan HIV-1 test (Roche Diagnostics GmbH, Grenzach-Wyhlen, Germany). Both assays are accredited in vitro nucleic acid amplification tests of *gag* for the quantitation of human HIV-1 RNA and both determine the results in copies/mL. The limit of agreement between the two assays is reported to be 0.126 copies/ mL 38. The detection limit was 20 copies/800 μL pure BAL fluid. For analysis, values below the detection limit were assigned 19 copies/800μL BAL fluid. These values are specifically marked in the figures. To allow direct comparison between viral load in serum and BAL fluid, the dilution factor of the BAL procedure was assesed by the urea method 39. Urea was measured by BUN Flex reagent cartridge (DF21, Siemens Healthcare Diagnostics GmbH, Eschborn, Germany). All data refer to copies/mL alveolar lining fluid, for easier reading the term copies/mL BAL will be used throughout the text.

### Flow cytometry

For phenotypic analysis freshly isolated PBMCs and BALMCs were stained with the surface marker antibodies (BD Biosciences, Johannesburg, South Africa) anti-CD3 Pacific Blue (UCTH1), anti-CD4 Alexa Fluor 700 (RPA-T4), anti-CD8 PerCP-Cy5.5 (SK1), anti-CD184 allophycocyanin (12G5/CXCR4) and anti-CD195 PE (2D7/CCR5). Staining and acquisition was performed as previously described 3. The flow cytometric gating strategy is illustrated in the Supporting Information Fig. 1. Photomultiplier voltage settings were not changed in between group comparison. Data analysis was performed with FlowJo software version 9.2 (TreeStar, Ashland, TX, USA).

### mRNA analysis

To estimate constitutive transcript abundance RNA samples were extracted immediately after BALMCs isolation. To compare the differences in transcript abundance threshold cycle (CT) values for β-actin were subtracted from the CT values of the gene of interest. To analyse *M. tuberculosis* induced changes in transcript levels, 5 × 10^5^ BALMC were cultured for 24 h in a 24-well plate with 1 mL RPMI and 10% heat-inactivated fetal calf serum (Gibco, Mowbray, South Africa) in the presence or absence of *M. tuberculosis* H37Rv at MOI 1:1. RNA isolation and quantitative RT-PCR were performed as previously described 40. The fold induction of genes was calculated by the ΔΔCT method (ΔCT in the presence of H37Rv minus ΔCT in the absence of H37Rv in culture) and values normalised by power 2*^x^* transformation. RANTES, MIP-1α and MIP-1β primers and probes were obtained from Applied Biosystems (Foster City, CA, USA).

### Multiplex bead array

MIP-1α, MIP-1β, RANTES and SDF-1α levels in serum and BAL fluid were assayed in batches by multiplex bead array (Bio-Rad Laboratories, Munich, Germany). Serum testing was performed according to the manufacturer’s instructions (User Bulletin #10014905 Rev C, download from http://bio-rad.com/bioplex). 500 μL BAL fluid was incubated with multiplex beads, 1% bovine serum albumin (Sigma-Aldrich, Steinheim, Germany) and protease inhibitor (Roche, Mannheim, Germany) on a roller device at 4°C overnight. BAL fluid volume was subsequently reduced on the 96-well Biorad plate with vacuum manifold. Samples were read on the Biorad Luminex reader using Bioplex manager 4.1 software. Chemokine levels in BAL fluid were normalised by the urea method as pg/mL alveolar lining fluid, which for easier reading is reported as pg/mL BAL throughout the manuscipt.

### Data analysis

Due to relatively small cell numbers in individual samples, not all analyses could be performed on all subjects. Statistical tests between groups were performed by the Mann–Whitney U-test, for paired data with the Wilcoxon Signed Rank test and for 2 × 2 tables Fisher’s exact test of probability. Non-parametric correlation was assessed by Spearman coefficient, association between normally distributed data was tested by Pearson’s correlation test. Comparison in figures are indicated as *p < 0.05; **p < 0.005 and ***p < 0.0005.

## Abbreviations

ART: anti-retroviral therapy
BAL: bronchoalveolar lavage
BALMC: BAL mononuclear cell
CCR5: beta chemokine receptor 5
CFP-10: culture filtrate protein 10
CT: threshold cycle
CXCR4: alpha chemokine receptor 4
ESAT-6: early secreted antigenic target 6
LDL: lower detection limit
SDF-1: stromal cell-derived factor 1

## Notes

As a service to our authors and readers, this journal provides supporting information supplied by the authors. Such materials are peer reviewed and may be re-organized for online delivery, but are not copy-edited or typeset. Technical support issues arising from supporting information (other than missing files) should be addressed to the authors.

Figure S1 Flow cytometric gating strategy of CCR5 and CXCR4 receptor expression on CD4^+^/CD8^+^ BALMCs. (A) The gating strategy used to identify CD4^+^ and CD8^+^ T cells from BAL is shown in representative density plots from a single person. From left to right, lymphocytes were selected using forward scatter/side scatter-area, subsequently followed by gating on CD3^+^ T cells and thereafter selecting CD4^+^ and CD8^+^ T cells. (B–E) Representative histograms show (B, D) CCR5 and (C, E) CXCR4 expression on (B, C) CD4^+^ and (D, E) CD8^+^ BALMCs. The grey histograms give one representative example of an HIV-1-uninfected person, the black histograms show one representative example of an HIV-1-infected person. The same strategy was used for phenotyping of PBMCs.
