# Supplementary material for: Relationship between chemokine receptor expression, chemokine levels and HIV-1 replication in the lungs of persons exposed to Mycobacterium tuberculosis
Source: Eur J Immunol. 2012 Dec 13;43(2):540–9. doi: 10.1002/eji.201242804 (PMC3791514; doi:10.1002/eji.201242804)

European Journal of Immunology

Supporting Information

for

DOI 10.1002/eji.201242804

Barbara Kalsdorf, Keira H. Skolimowska, Thomas J. Scriba, Rod Dawson, Keertan Dheda, Kathryn Wood, Jessica Hofmeister, Willem A. Hanekom, Christoph Lange and Robert J. Wilkinson

Relationship between chemokine receptor expression, chemokine levels and HIV-1 replication in the lungs of persons exposed to *Mycobacterium tuberculosis*

© 2012 WILEY-VCH Verlag GmbH & Co. KGaA, Weinheim

Relationship between chemokine receptor expression, chemokine levels and HIV-1 replication in the lungs of persons exposed to *Mycobacterium tuberculosis*

Barbara Kalsdorf[1,2], Keira H. Skolimowska[1,3], Thomas J. Scriba[4], Rod Dawson[5], Keertan Dheda[5], Kathryn Wood[1], Jessica Hofmeister[2], Willem A. Hanekom[1,4], Christoph Lange[2], and Robert J. Wilkinson[1,3,5,6]

Supporting Figure 1: Flow cytometric gating strategy of CCR5 and CXCR4 receptor expression on $CD4^+$ / $CD8^+$ BALMCs. (A) The gating strategy used to identify $CD4^+$ and $CD8^+$ T cells from BAL is shown in representative density plots from a single person. From left to right, lymphocytes were selected using forward scatter/side scatter (FSC/SSC)-area, subsequently followed by by gating on $CD3^+$ T cells and thereafter selecting $CD4^+$ and $CD8^+$ T cells. (B-E) Representative histograms show (B, D) CCR5 and (C, E) CXCR4 expression on (B, C) $CD4^+$ and (D, E) $CD8^+$ BALMCs. The grey histograms give one representative example of an HIV-1-uninfected person, the black histograms show one respresentative example of an HIV-1-infected person. The same strategy was used for phenotyping of PBMCs.

Supporting Figure 1

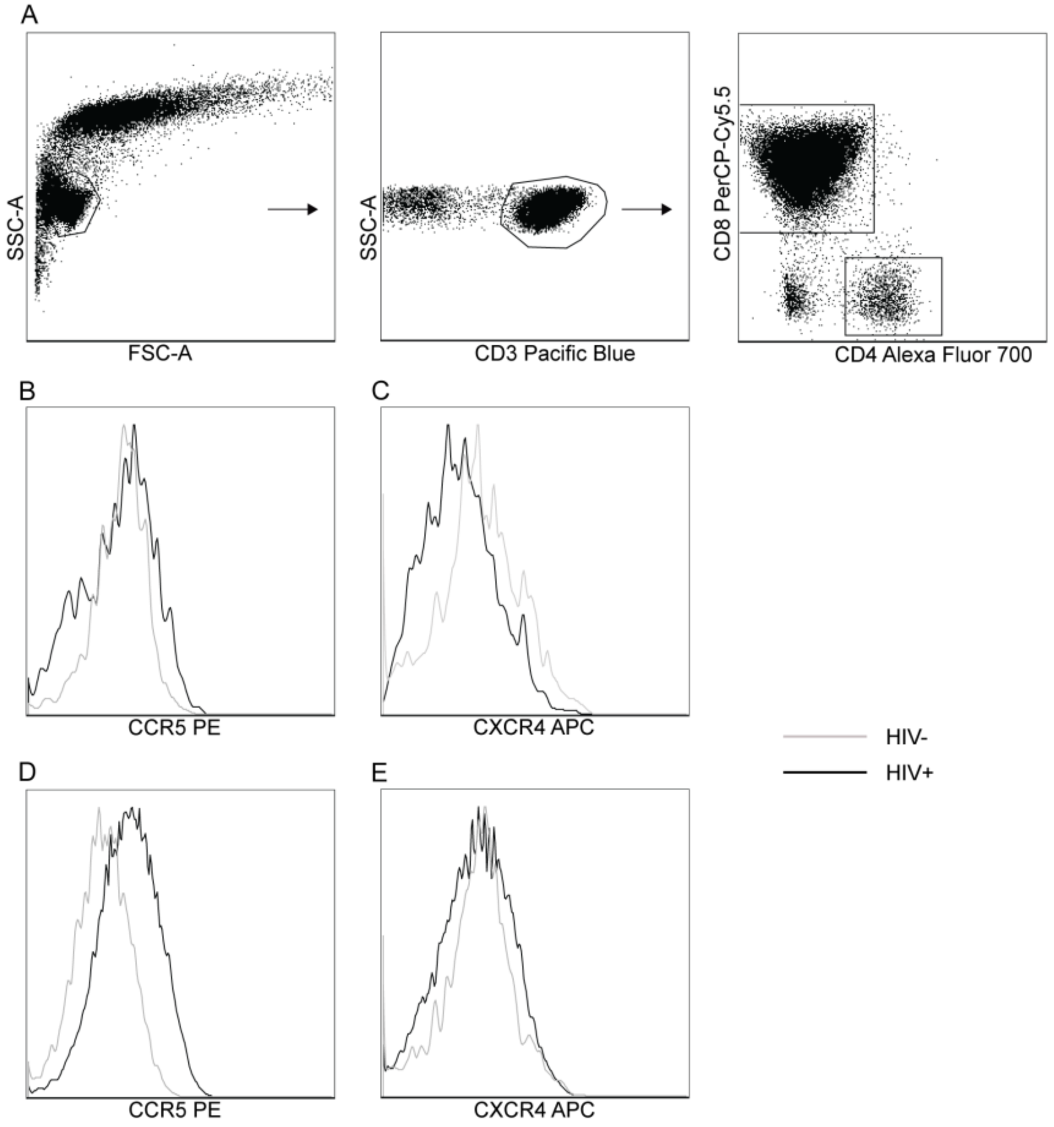

Supplement: Figure S1 — Flow cytometric gating strategy of CCR5 and CXCR4 receptor expression on CD4+/CD8+ BALMCs. (A) The gating strategy used to identify CD4+ and CD8+ T cells from BAL is shown in representative density plots from a single person. From left to right, lymphocytes were selected using forward scatter/side scatter-area, subsequently followed by gating on CD3+ T cells and thereafter selecting CD4+ and CD8+ T cells. (B–E) Representative histograms show (B, D) CCR5 and (C, E) CXCR4 expression on (B, C) CD4+ and (D, E) CD8+ BALMCs. The grey histograms give one representative example of an HIV-1-uninfected person, the black histograms show one representative example of an HIV-1-infected person. The same strategy was used for phenotyping of PBMCs. [file eji0043-0540-sd1.pdf]
